# 1793. Evaluation of Ambulatory Antibiotic Use in Children and Adult Patients of a Healthcare System in Bolivia Using the AWaRe Classification

**DOI:** 10.1093/ofid/ofac492.1423

**Published:** 2022-12-15

**Authors:** Rodolfo E Quiros, Elvio D Escobar, Juan Carlos Tapia, Sara E Cosgrove, Valeria Fabre

**Affiliations:** Sanatorio Las Lomas, Buenos Aires, Buenos Aires, Argentina; Clinica Foianini, Santa Cruz de la Sierra, Santa Cruz, Bolivia; Clinica Foianini, Santa Cruz de la Sierra, Santa Cruz, Bolivia; Johns Hopkins University Department of Medicine, Baltimore, Maryland; Johns Hopkins University, Baltimore, Maryland

## Abstract

**Background:**

The World Health Organization (WHO) has established the AWaRe (*Access*, *Watch* and *Reserve*) classification based on the impact of different antibiotics on antimicrobial resistance. For ambulatory patients, WHO recommends *Access* antibiotics to represent >60% of all prescriptions, and an *Access* to *Watch* (AW) index of ≥1.5. The use of *Access* and *Watch* antibiotics has not been evaluated in ambulatory patients in Latin America. The aim of this study was to describe antibiotic use among members of two health plans within a private health insurance in Bolivia.

**Methods:**

We retrospectively evaluated antibiotic use among 8,405 members of a private healthcare system in Santa Cruz de la Sierra, Bolivia, between Jan-2017 and Dec-2018. Antibiotic use was calculated as defined daily doses (DDD) per 1,000 member-days, and compared between two plans, one a Health Maintenance Organization (HMO) (2,419 members) and the other a Preferred Provider Organization (PPO) (5,986 members). In the HMO plan, members have a general practitioner (GP) as the primary point of care, whereas in the PPO plan, members can access specialists directly without a referral from the GP. Differences between groups were calculated using Byar test with 95% confidence intervals (CI).

**Results:**

Antibiotic use in DDD/1,000 member-days for the study period was 8.31 in ambulatory, 10.7 in the Emergency Department, and 0.72 in the hospital. Ambulatory antibiotic use in the HMO plan was lower than the PPO plan (6.95 vs. 8.89 DDD/1,000 member-days; diff. –1.94, 95%CI –2.15 to –1.72) (Table). Of all ambulatory antibiotics, 55% were *Access* and 45% were *Watch* (AW index: 1.24). By patient group, 52% of pediatric and 58% of adult prescriptions were *Access*; diff. –6%; p< 0.001 (AW index: 1.07 and 1.39, respectively). By health plan, 62% of HMO and 53% of PPO antibiotics were *Access;* diff. 9%; p< 0.001 (AW index: 1.13 and 1.67, respectively).

**Figure:**
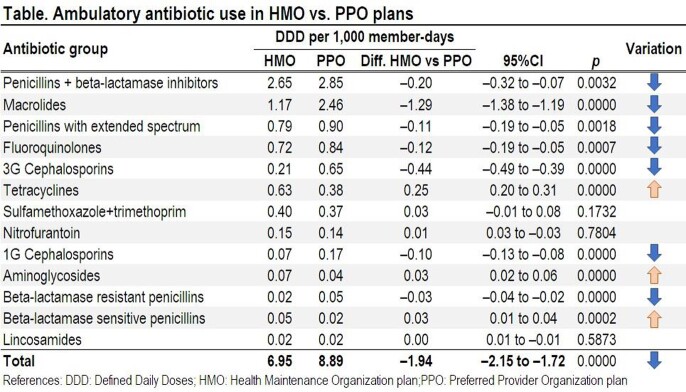

**Conclusion:**

Ambulatory antibiotic use in this cohort was high. Overall use of *Access* antibiotics was lower than the recommended by WHO; however, we found less broad-spectrum antibiotic use in the HMO plan compared to the PPO plan. These findings highlight the urgent need for antibiotic stewardship in the ambulatory setting, and the important role of GPs in appropriate antibiotic prescribing.

**Disclosures:**

**Sara E. Cosgrove, MD**, Basilea: Member of Infection Adjudication Committee.

